# Clinical perspectives of PSMA PET/MRI for prostate cancer

**DOI:** 10.6061/clinics/2018/e586s

**Published:** 2018-09-17

**Authors:** Felipe de Galiza Barbosa, Marcelo Araújo Queiroz, Rafael Fernandes Nunes, José Flávio Gomes Marin, Carlos Alberto Buchpiguel, Giovanni Guido Cerri

**Affiliations:** I Departamento de Radiologia, Hospital Sirio-Libanes, Sao Paulo, SP, BR; II Instituto de Radiologia, Hospital das Clinicas HCFMUSP, Faculdade de Medicina, Universidade de Sao Paulo, Sao Paulo, SP, BR

**Keywords:** Prostate Cancer, Positron Emission Tomography/Magnetic Resonance Imaging, Diagnostic Imaging, Tumor Staging, Local Neoplasm Recurrence

## Abstract

Prostate cancer imaging has become an important diagnostic modality for tumor evaluation. Prostate-specific membrane antigen (PSMA) positron emission tomography (PET) has been extensively studied, and the results are robust and promising. The advent of the PET/magnetic resonance imaging (MRI) has added morphofunctional information from the standard of reference MRI to highly accurate molecular information from PET. Different PSMA ligands have been used for this purpose including ^68^gallium and ^18^fluorine-labeled PET probes, which have particular features including spatial resolution, imaging quality and tracer biodistribution. The use of PSMA PET imaging is well established for evaluating biochemical recurrence, even at low prostate-specific antigen (PSA) levels, but has also shown interesting applications for tumor detection, primary staging, assessment of therapeutic responses and treatment planning. This review will outline the potential role of PSMA PET/MRI for the clinical assessment of PCa.

## INTRODUCTION

Prostate cancer (PCa) faces a new era of diagnosis and management with the use of next-generation imaging modalities based on positron emission tomography (PET). The advent of prostate-specific membrane antigen (PSMA) PET tracers has increased the clinical application of imaging diagnosis of PCa in different clinical settings, such as for primary staging, detection of biochemical recurrence (BCR), assessment of therapeutic responses and treatment planning. In Brazil, there were an estimated 61200 new cases of PCa in 2016, making PCa the second most prevalent neoplasia among men throughout the country and the third most common cause of cancer-associated mortality in men in Western countries 1,2.

PSMA is a type II transmembrane glycoprotein that is highly expressed in almost all PCa cells, with only 5-10% of primary PCa not having PSMA expression 3. PSMA PET positivity has been shown to increase with higher tumor stage and grade, higher prostate-specific antigen (PSA) levels and doubled time 4. Several studies have addressed the clinical impact of PSMA PET for PCa assessment, for primary staging, restaging/biochemical recurrence, radiotherapy planning and systemic therapy planning in patients who have previously undergone salvage treatment 5-8. Only one PSMA ligand has been approved by the Food and Drug Administration (FDA), the radiolabeled anti-PSMA antibody capromab pendetide (ProstaScint), which has a low accuracy for PCa detection, as it is a large antibody that binds to the intracellular domain of PSMA 9. On the other hand, there are a few small molecules PSMA ligands that bind to the active site in the extracellular domain of PSMA thus providing increased tumor uptake and high image quality 3.

The two first PSMA agents for PET imaging were ^18^F-DCFBC and ^68^Ga-PSMA-11, followed by another two probes with theranostic capabilities, the chelator-based PSMA-617 and the PSMA inhibitor for imaging and therapy PSMA-I&T. Later, some second-generation ^18^F-labeled PSMA ligands were introduced to overcome the high blood-pool activity and low tumor-to-background ratios of ^18^F-DCFBC, namely, ^18^F-DCFPyL and, more recently, ^18^F-PSMA-1007, which has very low urine clearance 3,9. All of these PET agents have already been translated to clinical applications and some have been used in PET/magnetic resonance imaging (MRI) systems.

The combination of structural, multiparametric functional, and molecular information of PSMA PET/MRI might represent a breakthrough for PCa evaluation. PET/MRI has shown superiority over PET/CT using a ^68^Ga-labeled PSMA for the detection of recurrent PCa; however, this modality has limitations regarding scatter correction 10. Some case reports have shown this potential advantage of PET/MRI in different clinical scenarios, such as for the diagnosis of primary tumors 11,12. It is believed that the use of PET/MRI might facilitate biopsy targeting, prediction/monitoring of tumor aggressiveness (especially for active surveillance) and radiation therapy planning (e.g., boost for a dominant intraprostatic lesion) 12.

For the proper clinical use of PET/MRI using PSMA agents, the workflow must be strictly optimized; an adequate protocol must be defined to avoid redundant information and long acquisition times. Nevertheless, it is mandatory to include high-resolution T2-weighted sequences combined with diffusion-weighted imaging (DWI) and perfusion on the MRI portion of the scan. The standard PET scan lasts 60 minutes, and there is an arguable advantage of performing either an early or a late acquisition.

This review will highlight the clinical perspectives of the use of PET/MRI with PSMA ligands for evaluating PCa including local detection, primary staging, identification of BCR, assessment of therapeutic responses and therapy planning.

### Local detection and primary staging

More than 90% of primary PCa lesions show moderate to high PSMA expression levels on PSMA PET 13, and many current studies have indicated that PET/MRI could be the single ideal imaging modality for staging PCa patients 10,14,15. There are already proposed protocols that are potentially viable for routine clinical application that have Prostate Imaging Reporting and Data System (PI-RADS) 2.0-compliant multiparametric MRI acquisitions of the prostate bed and whole-body PSMA PET 15.

The MRI component is the imaging modality of choice for precise morphologic evaluation and has higher spatial resolution and provides clearer anatomic delineation of the prostatic fossa and surrounding anatomical structures than PET 16, while PSMA PET is the superior modality for detecting metastases to the locoregional and extrapelvic lymph nodes, bones and visceral organs 17.

**Figure 1:**
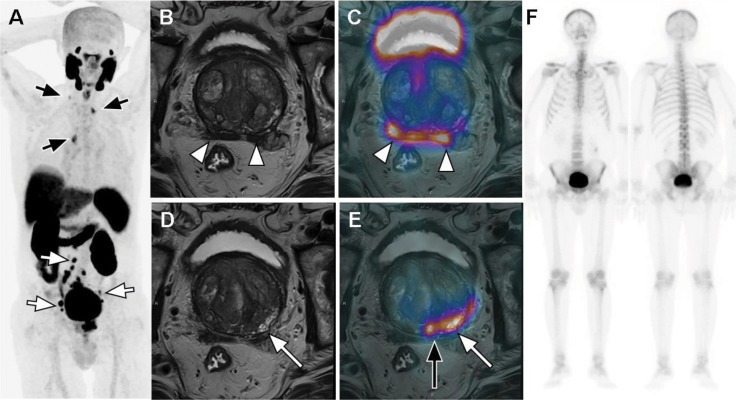
PSMA PET/MRI for initial staging of a 77-year-old high-risk PCa patient with a Gleason score of 7 (3+4) and a PSA of 44.0 ng/dL. The staging would be T3bN1M0 if the patient was assessed with conventional imaging, namely, mpMRI (B and D) and BS (F). PSMA PET (coronal maximum intensity projection (MIP)inf A and fused PET/MR in C and E) clearly depicts more extensive pelvic (white arrows in A), extrapelvic thoracic and right inferior cervical nodal involvement (black arrows in A), upstaging the patient to T3bN1M1a. The incremental value of multimodal assessment is also seen in terms of the patient's T status. There is concordance between PSMA PET and MRI regarding bilateral seminal vesicle involvement encompassing the whole right seminal vesicle and the medial portion of the left seminal vesicle (white arrowheads in B and C), whereas PSMA PET/MRI revealed a more extensive (black long arrow in E) primary lesion in the left and median peripheral zones of base and midgland than mpMRI (white long arrows in D and E).

Consequently, PSMA PET/MRI yields greater diagnostic accuracy for PCa localization than multiparametric MRI (mpMRI) or PET alone. These encouraging data indicate that hybrid imaging using morphologic, functional, and molecular information enhances diagnostic performance in patients with PCa for TNM staging 14 (Figure 1).

#### T Staging

MRI brings valuable conveniences over PET/CT due to the high soft tissue contrast and offers the advantages of functional MRI techniques 16. In turn, PSMA PET has a very specific molecular imaging target for PSMA-expressing tumors. Each imaging modality alone is capable of identifying tumor sites that would otherwise be missed or considered negative by the other technique. Thus, PSMA PET/MRI has higher sensitivity (76%) than either method used alone (58% and 64%) 14.

Diffusion-weighted and dynamic contrast-enhanced (DCE) MRI have potential to furnish biological characterization of tumor aggressiveness in PCa patients. Recent data have associated quantitative MRI parameters with Gleason score and tumor angiogenesis 18. Likewise, studies have indicated that the intensity of radiotracer accumulation in the primary tumor is correlated to PSA levels and Gleason score (i.e., the higher the PSA levels, Gleason scores and d'Amico risk scores are, the greater the PSMA uptake on PET) 19,20. The sum of these parameters could provide details about distinct tumor aggressiveness in different regions throughout the prostate gland and even within the same lesion. Accordingly, aggregating information regarding PSMA uptake, lesion cellularity, vascular permeability and contrast media kinetics has brought about rich data for tumor characterization 21. This is of particular relevance when considering the potential role of PSMA PET/MRI as a prebiopsy diagnostic tool that can be used to guide sampling of the most aggressive sites. Hence, this method could be indicated for detecting intraprostatic malignant lesions in untreated patients with newly diagnosed PCa 20.

Regarding evaluation of tumor extent and extracapsular and seminal vesicle invasion, studies have shown promising results with PSMA PET. These parameters are major concerns for treatment planning. Curative surgery is possible when none of these findings is present. Nerve-sparing surgical techniques might not be performed in men with extracapsular extension, leading to increased risk of urinary incontinence and erectile dysfunction after prostatectomy, mainly when a bilateral technique is employed. These factors also have a profound impact on prognostication, since extracapsular extension and seminal vesicle invasion are both related to an increased risk of recurrence and lymph node and bone metastases. PSMA PET/MRI can have an important impact for local staging of PCa prior to radical prostatectomy 22.

Finally, addition of the PET component could address two relatively common shortcomings of MRI by reducing the limitation of evaluating patients who have recently undergone recent biopsies and increasing the performance for assessing transition zone (TZ) lesions. With regard to the first issue, whereas functional MRI techniques, such as DCE and DWI, could potentially be flawed with pitfalls and artifacts due to prior biopsy, PSMA PET does not appear to be impaired by those issues 21. Regarding the second matter, imaging of cancer lesions in the TZ may be confounded with other conditions on MRI, especially benign prostatic hypertrophy. While the peripheral zone is the most common site of PCa where 75–85% of lesions are located, as many as 25% of patients might develop tumors in the TZ. Hence, tumors in the TZ of the prostate are often missed by MRI alone, increasing the need for an additional method of detecting PCa in this location, and this need can be fulfilled by identifying the presence or absence of PSMA uptake 23.

Multimodal evaluation might facilitate biopsy orientation and lead to an impact on management, especially for predicting and monitoring tumor aggressiveness during active surveillance, determining more important targets when planning radiation therapy (dose escalations within a prostate clinical target volume) 24 and planning appropriate surgical technique and intraoperative management.

#### N Staging

PSMA PET is decidedly superior to MRI in terms of identifying distant metastases in patients with intermediate to high-risk PCa. As the method becomes more present in clinical settings, presumably many patients who are staged as N0 or M0 by current imaging evaluation will be more accurately staged as N1 or M1 6,24. Pretreatment staging with PSMA PET has the potential to be established as the standard of care for imaging in these patients since the success of therapy relies on precise inclusion of involved sites would remain untreated when staging with conventional imaging 24.

Involved lymph nodes are diagnosed by MRI when morphologic changes such as enlargement or round shape are present, while PSMA PET can demonstrate metastasis based on tracer uptake in morphologically unremarkable lymph nodes as small as 2 mm 13, even though this modality is also influenced by nodal size 25. A recent study reported that PSMA PET scans revealed previously unknown nodal involvement in 39% of the patients 6. Additionally, combination of PSMA PET with mpMRI is a promising path for improving the capabilities of PET to the greatest extent and ultimately resulting in better determination of nodal status 24. A recent template-based analysis study including 130 patients revealed that the sensitivity, specificity and accuracy of PSMA PET were 68.3%, 99.1% and 95.2%, respectively, while for morphological imaging the sensitivity, specificity and accuracy were 27.3%, 97.1% and 87.6%, respectively 13.

This is of paramount importance when curative local treatment of the prostate is considered, especially for planning external radiation therapy and surgical resection. With PSMA PET, a large number of patients may benefit from dramatic changes in the contouring of the targeted volumes and in the prescribed dose of radiation therapy. PSMA positive nodes tend to receive higher doses than the adjacent pelvic nodal volumes. Additionally, clinical target volume (CTV) can be extended to treat areas of disease that were not identified by conventional studies or targeted by consensus CTVs 24.

#### M Staging

In intermediate and high-risk PCa patients, the current preoperative staging for bone metastases includes MRI/CT and bone scintigraphy.

As ^99m^Tc-MDP displays osteoblastic activity, this modality demonstrates uptake in areas of degenerative and inflammatory diseases and fractures resulting in a low specificity for metastases 26. A recent investigation including 126 patients revealed a sensitivities and specificities for secondary osseous involvement of 98.7-100% and 88.2-100%, respectively, for PSMA PET and 86.7-89.3% and 60.8-96.1%, respectively, for bone scan (BS) 27. Another recent study reported that PSMA PET scans revealed previously unknown distant metastatic disease in 16% of patients 6.

Assessment of whole-body osseous tumor burden has also been proposed in recent studies investigating the role of PSMA PET as an exploratory imaging technique for prognostication and evaluation of potential objective responses 28.

The primary therapy for metastatic disease is androgen deprivation therapy. However, it is controversial among many clinicians if a distinct treatment approach should be made for patients with limited metastatic disease (oligometastatic) compared with those with diffuse metastatic disease. Stereotactic radiosurgery and metastatectomy with or without androgen deprivation therapy are being investigated in numerous studies 29. Therefore, correct identification of these patients is gaining importance, and PSMA PET imaging has emerged as an important technique in this scenario 24.

Finally, other distant lesions including visceral and soft tissue metastases that can easily be missed by conventional investigation are potentially detectable with the whole-body technique; in particular, pulmonary 10,20, genital and soft tissue lesions can be detected with this method 20,30.

### Biochemical recurrence

PCa relapse after curative intent treatment (prostatectomy or radiation therapy) is defined as BCR, which is diagnosed by an increased PSA level. Up to 40% of patients develop BCR during their lifetime, and approximately 25% develop clinical recurrence after 7-8 years 31. Detecting specific BCR sites with imaging has been a challenging for the last 10 years, even with the development of dedicated MRI. Disease detection is clinically relevant because it can guide more effective treatment planning and consequently avoid futile systemic or localized treatment approaches and their related side effects 32.

PCa is a slowly recurrent disease in majority of patients; thus, detecting very low volume disease in the BCR scenario is important due to the possibility of salvage treatment (surgery or radiotherapy), postponement of systemic androgen deprivation treatment (ADT), or even cure 33. The urological community suggests that with this treatment approach patients may have prolonged and improved quality of life; however, long-term patient outcomes with this approach still need to be demonstrated.

Current conventional imaging modalities (bone scintigraphy and computed tomography (CT)) have low accuracy for pelvic node and bone metastasis in patients with low PSA levels 31. MRI has become the method of choice for local recurrence, with a sensitivity of approximately 75%. Although MRI performs better than conventional imaging, identifying local recurrence is the least important for making changes to salvage treatment, as radiation therapy of the prostate bed is the first indicated method for BCR in patients with low PSA levels 32.

**Figure 2:**
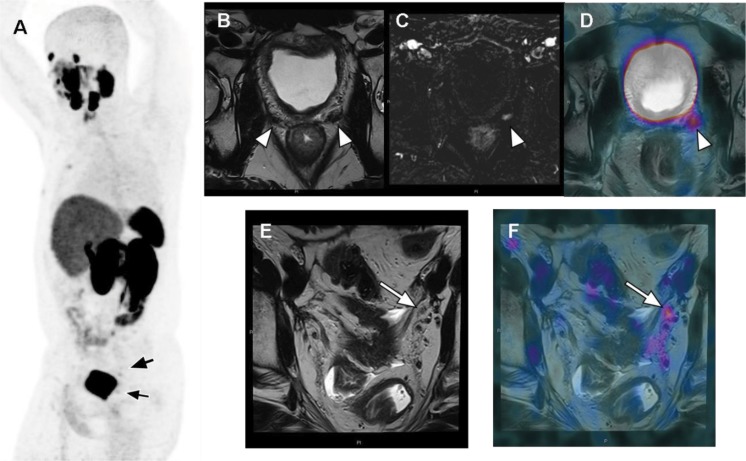
Evaluation of BCR in a 68-year-old patient treated with prostatectomy with a Gleason 7 adenocarcinoma (8 years) and prostate bed radiation (3 years), with a current PSA level of 0.29 ng/ml. ^68^Ga-PSMA-fused PET/MRI MIP images (A) demonstrated 2 pathologic areas of focal uptake in the pelvis (black arrows). Axial T2-weighted imaging (B) showed nonspecific bilateral hypointense tissue in the prostate bed (arrowheads); however, T1-weighted perfusion imaging (C) demonstrated hypervascularity in the left tissue (arrowhead), which also had focal PSMA uptake on fused PET/MR imaging (D), confirming local recurrence. Moreover, a 4-mm left external iliac lymph node (white arrow) was almost not visible on axial T2-weighted imaging (E) but had focal uptake on fused imaging (F), which was very suspicious for pelvic nodal recurrence.

PSMA PET/CT has become the breakthrough imaging method for PCa relapse in the last 5 years 34. The literature has demonstrated that PSMA has better sensitivity and specificity than conventional methods or choline PET for detecting tumor recurrence, especially in patients with low PSA levels (<1.0 ng/mL) 35,36. The sensitivity rates of PSMA PET/CT according to PSA levels are 55-60% (0.2-0.5 ng/mL), 72-75% (0.5-1.0 ng/mL), 93% (1.0-2.0 ng/mL) and 97% (≥2.0 ng/mL). A recent study of PET/MR showed a higher detection rate in patients with very low PSA levels than those of other PET/CT studies in the literature, with detection rates of 44% (<0.2 ng/mL) and 72.7% (0.2-0.5 ng/mL) 37. These findings can be explained by the higher sensitivity of PET detectors in PET/MRI system and/or due to higher resolution imaging in the prostate bed. Clinical parameters that can influence also detection of BCR are high PSA levels (>1.0 ng/mL), short PSA doubling time (<6 months) and high Gleason score (≥8), all of which increase the detectability rate of BCR on PSMA PET 4,38 (Figure 2).

PCa recurrence has a less predictable pattern of spread than imaging before PSMA. The most commonly detected sites of BCR are the abdominopelvic lymph nodes (50-55%), supradiaphagmatic lymph nodes (5.2%), bones (35.9%), local recurrence (35.1%) and other organs (e.g., lung, liver) with 5.2% 4. Different than was previously thought, nodal recurrence is more common than local recurrence and not only involves usual lymph node stations (obturator, external iliac, internal iliac and common iliac) but also involves atypical pelvic atypical. Mesorectal lymph node involvement is one of the most common atypical nodal stations in the pelvis, with 15.8% detection rate according to Hijazi et al. 39. As demonstrated in some studies 4,39, PSMA PET/CT can detect lymph nodes that measure less than 5 mm in the short-axis diameter, explaining its better sensitivity for nodal detection than CT or MRI. Regarding systemic spread of PCa to bone, few papers have demonstrated the clear superiority of PSMA PET for detecting bone lesions compared to bone scintigraphy (BS), and that BS did not have significant additional diagnostic value in BCR scenario 27,40,41. However, focal bone PSMA uptake alone should not be immediately considered metastasis; moreover, if moderate/mild uptake, which has to be correlated with CT morphology, is noted, it can decrease the possibility of false positives 40, as a few cases of benign lesions with PSMA uptake have been reported 42,43.

Literature has confirmed the abovementioned findings, which have deep clinical implications. As recommended in current guidelines, a negative conventional imaging evaluation suggests that a patient is suitable for salvage radiotherapy of the prostate bed; however, PSMA imaging could significantly change treatment decisions by detecting small pelvic lymph nodes or bone metastasis. Recent study by Hope et al. has shown that PSMA imaging had a significant impact for evaluating BCR in 53% of patients and led to major changes in management and avoided unnecessary imaging studies (BS, CT and MRI) and invasive procedures 5. In this study, most of the major changes involved conversion to targeted therapy (mainly radiotherapy) in 32% of patients or conversion to systemic treatment (mainly ADT) in approximately 10% of patients. Few studies have addressed better treatment plan based on salvage treatment in oligometastatic patients (with bone and/or nodal involvement) with pelvic lymphadenectomy 44 or PSMA-guided radiation therapy 45,46. Calais et al. 8 showed that even when pelvic lymph node stations were included in standard radiotherapy plans for salvage BCR < 1.0 ng/mL, 19% of patients had metastasis (nodal and/or bone) outside the radiation delineation area determined based on CT alone. A complementary study showed that when PSMA PET identified positive lymph nodes, the standard radiotherapy response was 62% compared to 85% when PSMA PET was negative for nodal involvement 47, demonstrating the possibility of generating tailored radiation plans guided by PSMA imaging.

Despite these promising results on the clinical impact of changing management based on PSMA PET evaluation of BCR, improvement in long-term outcomes must be demonstrated to confirm that this transformational molecular imaging technique is useful in clinical practice. As recently discussed in an editorial article published in the Journal of Nuclear Medicine 48, the major challenge oncologists face in managing PCa is identifying which patients harbor significant and nonsignificant recurrence. Current treatment options have rather low success in curing patients, and avoiding toxicity and promoting quality of life are fundamental objectives in treatment selection.

### Assessment of therapeutic response

Numerous therapeutic modalities are available for early or advanced PCa. Various options including local to systemic treatments can be used in a multitude of clinical scenarios. Furthermore, beyond the efficacy of each treatment modality, toxicities and costs play important roles in therapeutic decisions and adequate management of PCa, creating a demand for precise assessment of therapeutic benefits. In this setting, imaging can provide helpful information about residual disease extension, the degree/depth of response, biological heterogeneity/behavior of neoplasia, disease progression and even side effects 49. Therefore, assessing therapeutic responses in PCa is a complex issue, since each stage of disease has very specific features and requires different approaches. Localized disease can be treated with surgery or radiotherapy and followed by active surveillance with comparable outcomes 50. Advanced disease encompasses a large spectrum of tumoral phenotypes and clinical conditions, varying from favorable oligometastatic 29 presentations to disseminated metastatic disease; each situation requires different treatments, such as hormones, radiation or chemotherapy. However, both localized and advanced prostatic disease are under the influence of androgen receptor (AR) signaling, which is a crucial driving pathway for growth and proliferation of PCa cells and the main target for ADT 51. An important condition is metastatic castration resistant PCa (mCRPC), which is characterized by persistent AR stimulation independent of ADT 52 and is frequently associated with disseminated disease and a poor prognosis 53. In mCRPC, an important characteristic that favors imaging is that serum PSA, a consolidated surrogated marker of disease progression, can be dissociated from real burden of metastatic disease 49.

#### Traditional and newer imaging techniques

Traditionally, the effects of local therapies (i.e., radiotherapy) have been evaluated by MRI 54,55 (more in the context of suspicion of recurrence than for response evaluation), while systemic treatments are evaluated by CT (especially by means of the Response Evaluation Criteria in Solid Tumors (RECIST) 1.1 criteria) 56 and BS 57. However, despite the wide usage, good reproducibility and consolidated reimbursement of these approaches, they have several limitations including pitfalls for evaluating treated prostate glands, the low specificity of MRI 54, the low sensitivity of CT for normal sized lymph nodes and bone lesions, and drawbacks of BS, which are mostly related to its low spatial resolution, susceptibility to “flare” phenomenon and indirect measurement of bone metastatic activity (i.e., assessing only the reaction of normal bone to an injury caused by metastasis, leading to a loss of accuracy in assessment of therapeutic responses) 58.

The increasing availability of new imaging techniques and modalities has changed this landscape. By mixing anatomical and functional features (especially with the use of DWI), mpMRI has allowed a new perspective on predicting and evaluating responses after radiotherapy 59 and ADT for locoregional disease 55. BS methods for quantitatively evaluating the whole skeleton have allowed the development of biomarkers and added prognostic power to this commonly used methodology 60. More recently, whole-body MRI, based on DWI sequences, has been proposed for evaluating treatment responses in patients with bone metastases and has shown promising results 61; this technique has been included in recent standardization procedure guidelines 62 despite its low availability and some limitations related to low specificity 63. In this evolving scenario, PET imaging, as a relatively recently developed modality, has shown great value in PCa management. Despite some initial disappointment and lessons learned with use of FDG PET and PET/CT for evaluating PCa 64, precise comprehension of tumor biology and appropriate patient selection have provided valuable knowledge, allowing the rational use of this technology for selected indications 65, and, more importantly, have paved the way for molecular imaging with others tracers (i.e., ^18^F-choline and ^18^F/^68^Ga-PSMA) for PCa evaluation, specifically in assessment of therapeutic responses. Parallel to the development of new tracers t, the conception of new hybrid PET/MRI equipment has brought a new perspective of synergy of the strengths of each modality 66.

#### PSMA PET/MRI

How does the PSMA PET component of PSMA PET/MRI add value to the assessment of therapeutic responses?

**Figure 3:**
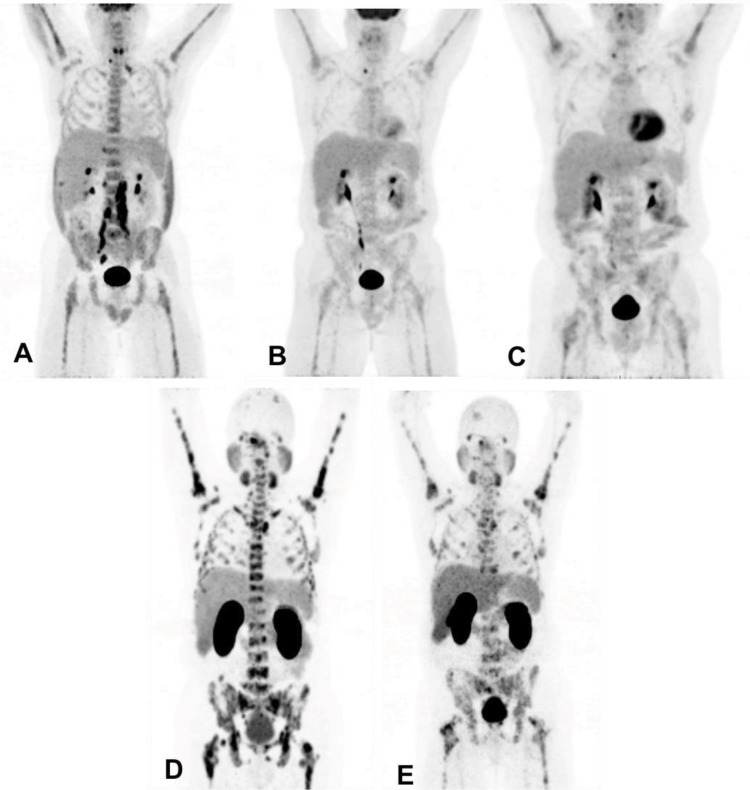
Example of different biological behaviors of PCa with treatment assessed by different tracers: (A) FDG PET in patient with advanced PCa showing retroperitoneal nodal and diffuse bone disease; (B) After beginning ADT, FDG PET shows a marked reduction in FDG uptake in the lesions; (C) PCa has become resistant to castration. Note subtle increase in diffuse FDG uptake in the skeleton. (D) At the same timepoint as C, PSMA PET was performed, showing marked bone disease, leading to an improved assessment of disease extension; and (E) PSMA PET performed 4 months after chemotherapy shows partial reduction in PSMA expression of diffuse skeletal involvement.

**Figure 4:**
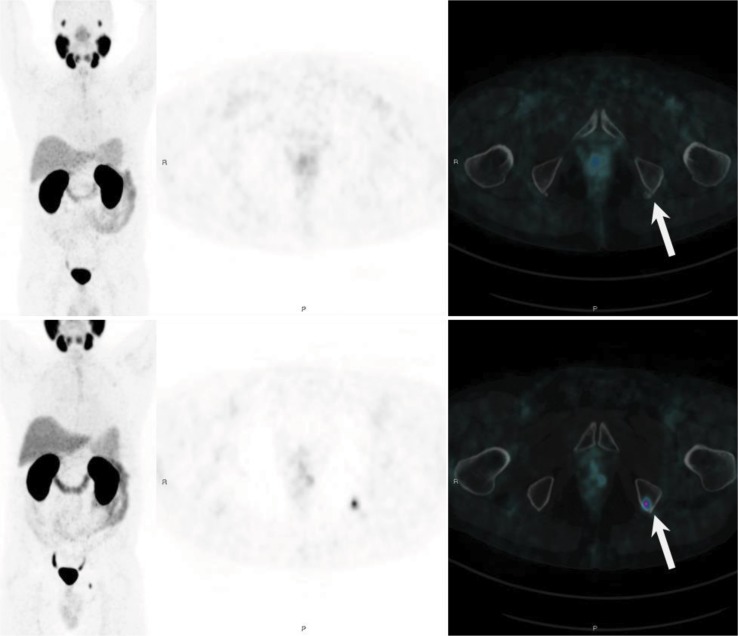
PSMA PET showing long-term PSMA expression against ADT in a patient with recurrent PCa who previously underwent pelvic radiation. The bottom row shows a bone metastatic lesion in left ischiatic tuberosity. The top row shows a new PSMA PET scan performed 10 months after beginning ADT, with resolution of the molecular expression of PSMA, probably reflecting a therapeutic effect of ADT.

PSMA is overexpressed in aggressive, poorly differentiated and metastatic PCa 67, a characteristic that confers enormous potential for using this molecular imaging marker to evaluate progression of disease. However, different from FDG, which appears to have predictable uptake and a positive correlation with AR status 68 of neoplastic cells (Figure 3), PSMA expression has some particularities, especially related to AR signaling and blocking of ADT 69. As addressed by previous preclinical 51,69 and clinical 67 reports, PSMA expression in PCa cells can rapidly increase after AR inhibition in hormone-sensitive PCa 69, leading to potential misinterpretation of PET/MRI or PET/CT scans for therapeutic assessment. On the other hand, therapeutic effect (i.e., reduction in the neoplastic cell population) along with ADT can be accurately assessed with PSMA PET several months after initiating therapy 70 (Figure 4), raising the unanswered question of when the turning point from initial PSMA overexpression to decreased overall expression due to reduction in the neoplastic cell population occurs. Given that ADT is one of the cornerstones of current PCa management, further studies are expected in this area.

For other treatment modalities, such as radiotherapy (including the growing indications for stereotaxic radiotherapy for oligometastases 71), chemotherapy 72 and radionuclide therapies (with ^177^Lu/^225^Ac-PSMA or ^223^Ra) 73, PSMA expression appears to be more directly linked with disease status and therapeutic effects, showing a good correlation with PSA values and emerging as a promising imaging tool and a potential prognostic biomarker.

As many reports using PSMA PET/MRI or PET/CT to assess treatment response have emerged, a critical issue needs to be addressed: standardization. Using established data for FDG, many authors have extrapolated the PET Response Criteria in Solid Tumors (PERCIST) criteria 74 to PSMA PET 71,72 with satisfactory but still very preliminary results. Like in FDG PET for other types of neoplasia, interesting whole-body quantification algorithms have been proposed for PSMA PET, and many of them are focused on calculating the burden of bone disease, its variations along during treatment and correlation with consolidated surrogated biomarkers (i.e., PSA) with exciting initial results 75. Given the prognostic importance of bone metastases in the natural progression of PCa, due to the a lack of robust criteria for evaluating the responses bone metastases and the limitations BS limitations, whole-body quantitative algorithms are a promising area for PSMA PET, particularly PSMA PET/MRI applications for PCa management.

How does the MRI component of PSMA PET/MRI add value to assessment of therapeutic responses?

The main focus of this paper is to discuss potential PSMA PET/MRI applications for PCa. The main strength of PSMA PET, as previously discussed, is powerful molecular *in vivo* tumor characterization with high sensitivity and specificity. MRI is a consolidated morphological imaging method that provides excellent characterization of anatomical and soft tissue contrast, and its strengths are not limited to this. MpMRI, which has incorporated functional sequences, has assumed a central role in PCa management. Functional sequences, mainly DWI, have high sensitivity (despite low specificity) for detecting and characterizing neoplastic tissue with localized or whole-body imaging, and have comparable performance to PET radiotracers for regions such as bone marrow 76. As with PET imaging, DWI requires quantification, mainly through apparent diffusion coefficient (ADC) maps.

In summary, relatively little early data are available regarding PSMA PET/MRI for assessment of therapeutic response in PCa. Despite the great potential of this modality and encouraging preliminary results, further trials are necessary to provide a better clinical understanding of PSMA expression behavior compared various therapies modalities (mainly ADT), to optimize molecular and functional response criteria and to improve both PET and MRI quantitative algorithms.

PSMA PET imaging represents an important breakthrough in molecular imaging for PCa evaluation. Regardless the PSMA ligand used, this emerging diagnostic modality offers substantial advantages over conventional diagnosis in different clinical settings. For lesion detection, PSMA PET/MRI might complement MRI alone for identifying clinically significant tumors and possibly for selecting target lesions in the clinical scenario of a persistent suspicion of PCa with a previously negative biopsy. At diagnosis, PSMA PET/MRI adds value to N and M staging, with notable clinical impact on patient management, especially in intermediate- and high-risk patients. In the context of BCR, PSMA PET/MRI has its most robust application, combining the molecular information from PET with the morphofunctional data from MRI, including the gold standard MR perfusion. Finally, PSMA PET imaging has been studied as an imaging biomarker of tumor responses with some encouraging but still preliminary results.

## AUTHOR CONTRIBUTIONS

Barbosa FG, Queiroz MA, Nunes RF and Marin JF made substantial contributions to the design of the work, drafting of the work, and final approval of the version to be published and agree to be accountable for all aspects of the work in ensuring that questions related to the accuracy or integrity of any part of the work are appropriately investigated and resolved. Buchpiguel CA and Cerri GG made substantial contributions to the design of the work, revised critically for important intellectual content, and gave final approval of the version to be published; they agree to be accountable for all aspects of the work in ensuring that questions related to the accuracy or integrity of any part of the work are appropriately investigated and resolved.
